# Supplementary material for: Light rain exacerbates extreme humid heat
Source: Nat Commun. 2024 Aug 26;15:7326. doi: 10.1038/s41467-024-51778-9 (PMC11347704; doi:10.1038/s41467-024-51778-9)
Supplement: Supplementary file 1 — Supplementary Information [file 41467_2024_51778_MOESM1_ESM.pdf]

Supplementary Information

Light Rain Exacerbates Extreme Humid Heat

Zhanjie Zhang[1], Yong Wang[1*], Guang J. Zhang[2*], Cheng Xing[3], Wenwen Xia[1,4] and Mengmiao Yang[5,6]

[1]Ministry of Education Key Laboratory for Earth System Modeling and Department of Earth System Science, Tsinghua University, Beijing, China

[2]Scripps Institution of Oceanography, La Jolla, CA, USA

[3]National Key Laboratory of Microwave Imaging Technology, Aerospace Information Research Institute, Chinese Academy of Sciences, Beijing, China

[4]State Key Laboratory of Numerical Modelling for Atmospheric Sciences and Geophysical Fluid Dynamics, Institute of Atmospheric Physics, Chinese Academy of Sciences, Beijing, China

[5]Key Laboratory for Humid Subtropical Eco-Geographical Processes of the Ministry of Education, Fujian Normal University, Fuzhou, China

[6]School of Geographical Sciences, Fujian Normal University, Fuzhou, China

Corresponding author: Y.W. (yongw@mail.tsinghua.edu.cn) and G. Z. (gzhang@ucsd.edu)

The PDF file includes:

Supplementary Figures 1 to 17

Supplementary Table 1

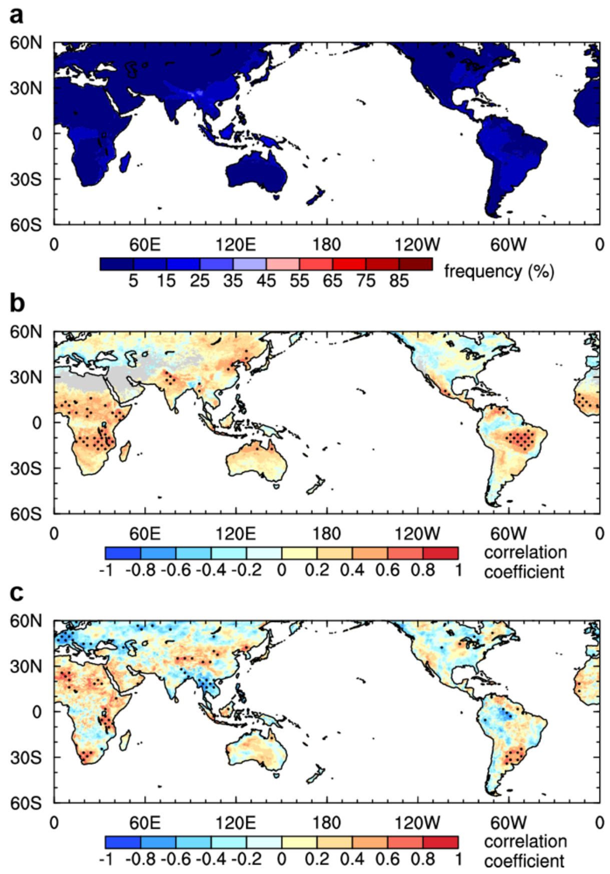

Supplementary Figure 1 | Rainfall and its relationship with wet bulb globe temperature (WBGT) during the hottest four months. a, Global distributions of occurrence frequency of daily rainfall larger than 20 mm d^{-1}. **b,** Temporal correlation coefficients between daily rainfall larger than 20 mm d^{-1} and WBGT. **c,** Temporal correlation coefficients between total precipitation and WBGT. Areas exceeding the 95% confidence level of the t-test in **b** and **c** are stippled.

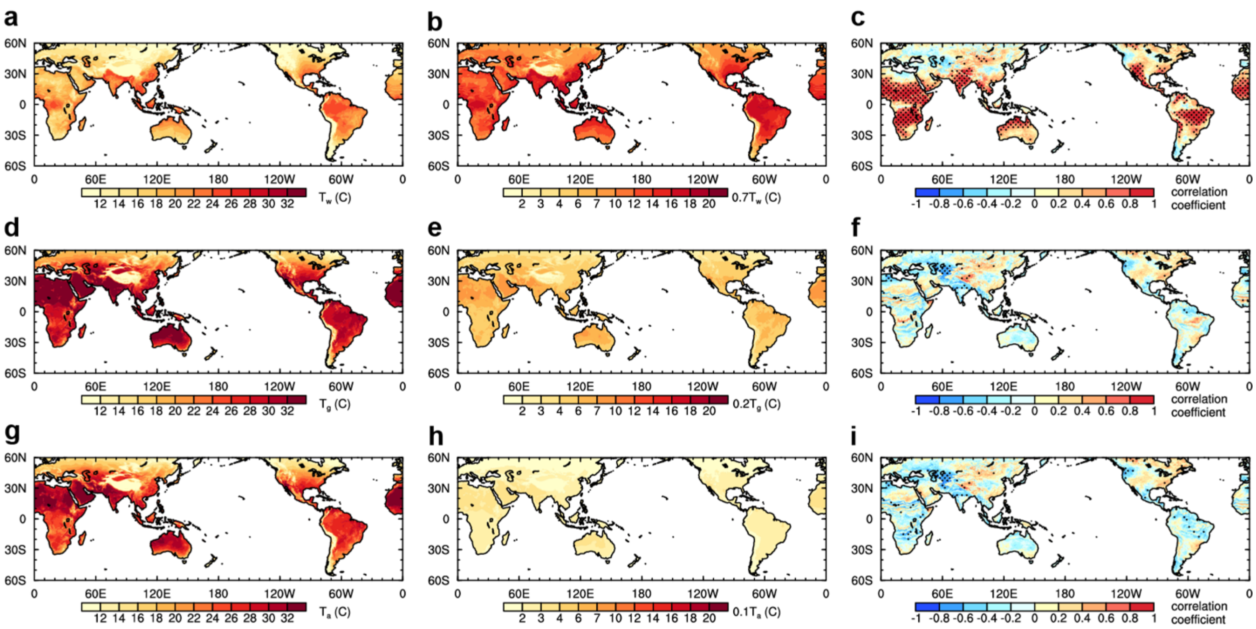

Supplementary Figure 2 | Wet bulb, black globe, and dry bulb temperatures, and their relationships with light rain frequency in the hottest four months. a, b, c, Intensity of natural wet bulb temperature (T_w) (**a**), 0.7 times T_w (**b**), and its correlation with light rain frequency (**c**). **d, e, f,** Intensity of black globe temperature (T_g) (**d**), 0.2 times T_g (**e**), and its correlation with light rain frequency (**f**). **g, h, i,** Intensity of dry bulb temperature (T_a) (**g**), 0.1 times T_a (**h**), and its correlation with light rain frequency (**i**).

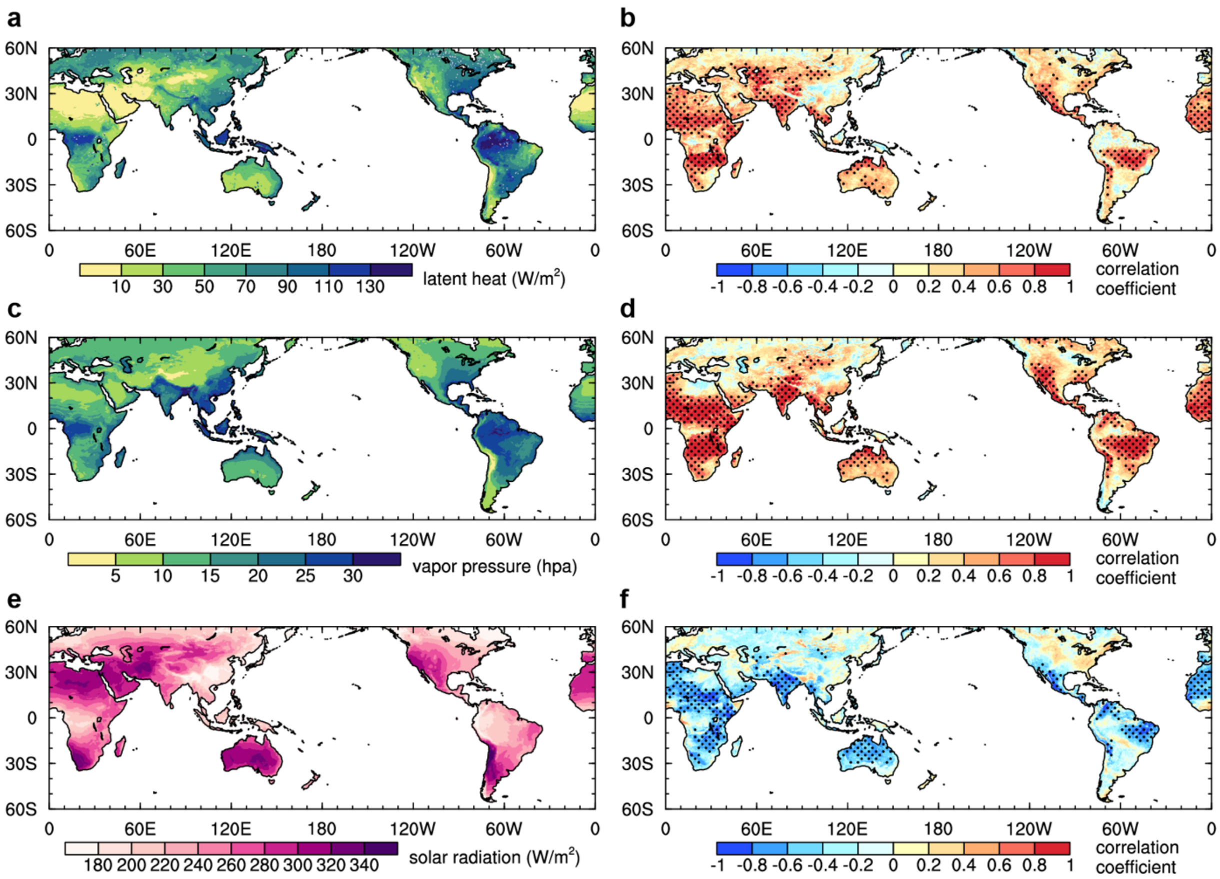

Supplementary Figure 3 | Surface latent heat flux, water vapor pressure, downwelling shortwave radiation, and their relationships with light rain frequency in the hottest four months. a, b, Intensity of surface latent heat flux (**a**) and its correlation with light rain frequency (**b**). **c, d,** Intensity of surface vapor pressure (**c**) and its correlation with light rain frequency (**d**). **e, f,** Intensity of surface downwelling solar radiation (**e**) and its correlation with light rain frequency (**f**).

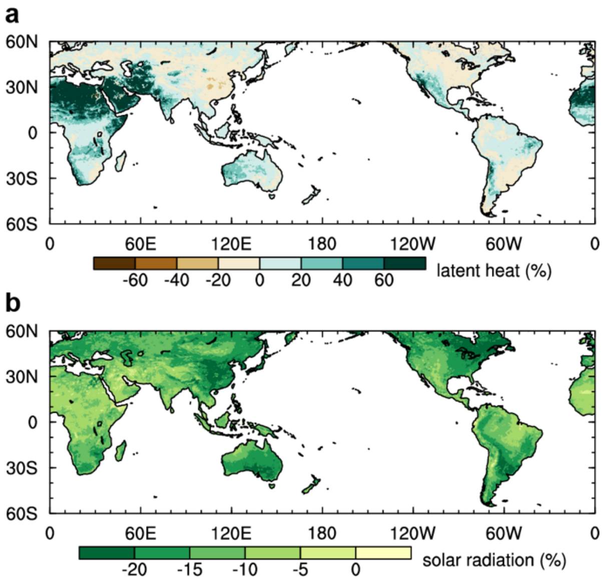

Supplementary Figure 4 | Changes on light rain days relative to non-rainy days. Changes in surface latent heat flux (**a**) and downwelling solar radiation (**b**) on light rain days relative to non-rainy days in the hottest four months.

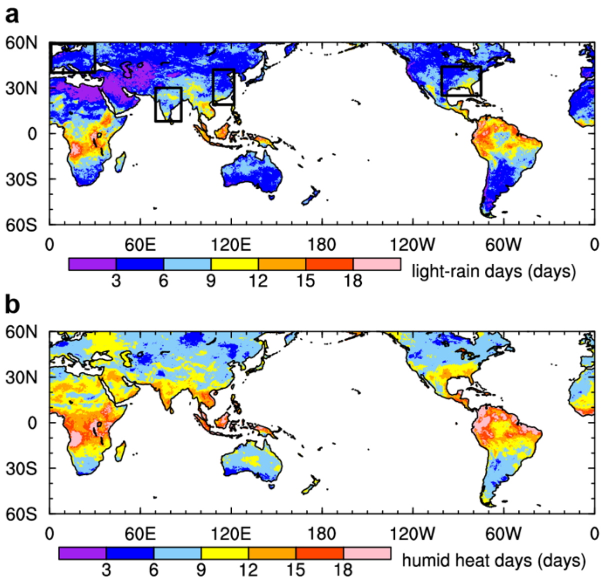

Supplementary Figure 5 | Inter-annual variation characteristics of the light rain days during the extreme humid heat days and the day before and of extreme humid heat days. The standard deviation of the annual number of light rain days on humid heat wave days and the day before (**a**) and of the annual number of humid heat wave days (**b**).

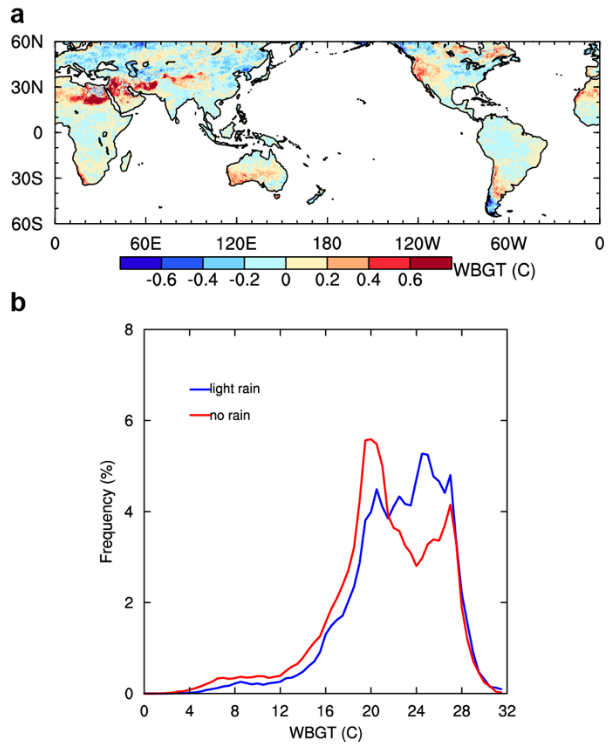

Supplementary Figure 6 | Wet bulb globe temperature (WBGT) intensity and frequency distributions of humid heat waves. a, The difference in the intensity of humid heat waves between light rain days and non-rainy days. **b,** The occurrence frequency of WBGT intensity of humid heat events for light-rain and non-rainy cases over (60°S −60°N) in the year 2001.

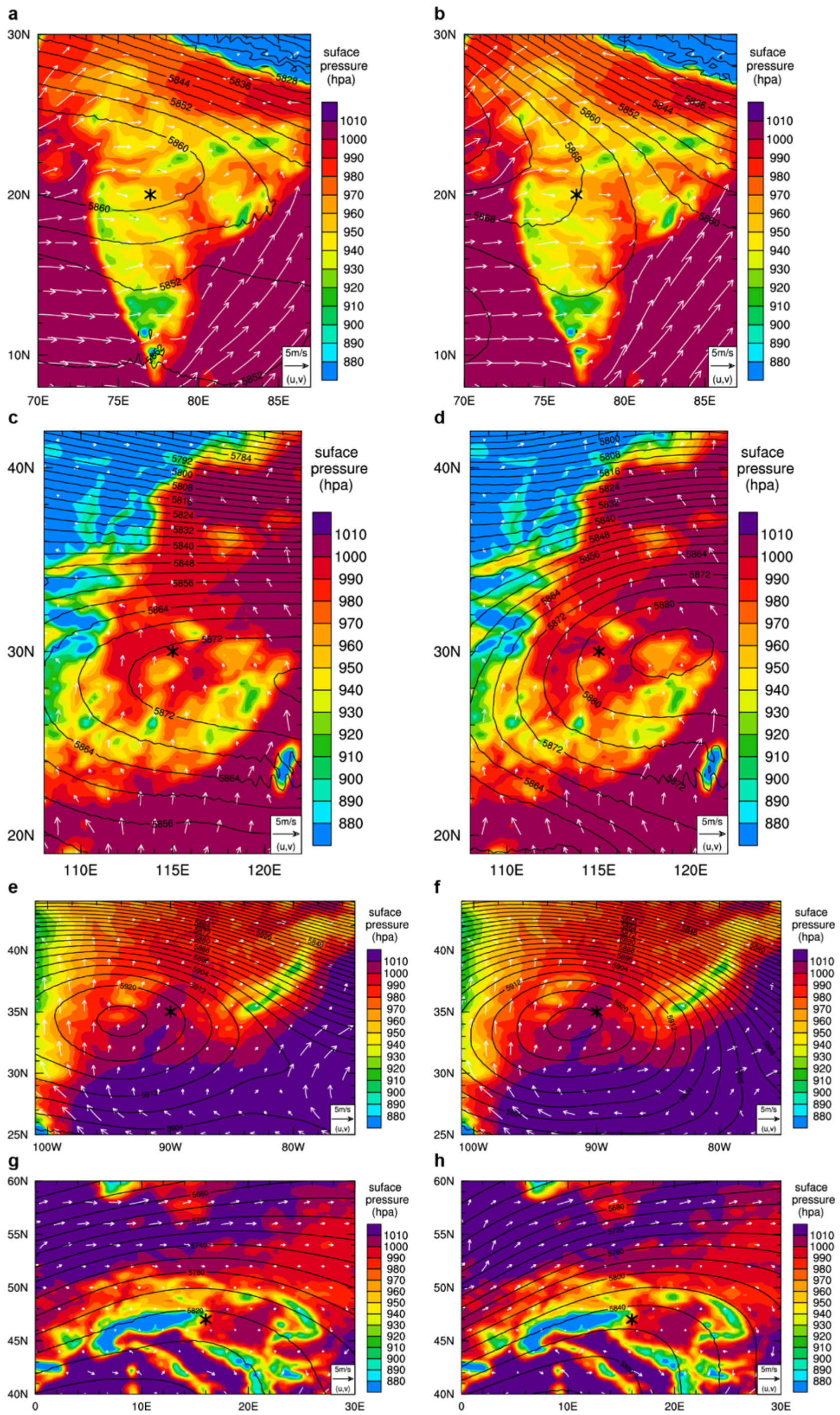

Supplementary Figure 7 | The background large-scale meteorological fields of light rain and no-rain humid heat waves. The composite fields of surface pressure (shadings), 500hpa geopotential height (contours) and 10m wind speed (vectors) during humid heatwaves with light rain (left) and without rain (right) occurring in the selected grid cells (asterisks) in India (**a, b**), eastern China (**c, d**), eastern United States (**e, f**) and Europe (**g, h**).

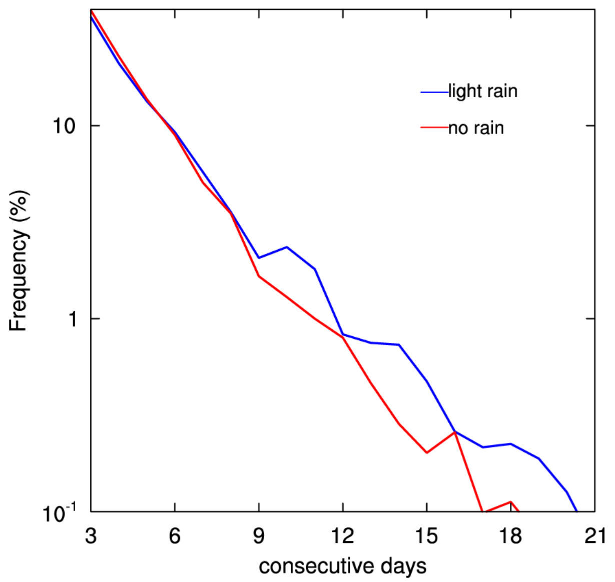

Supplementary Figure 8 | Frequency distributions of duration of humid heat events. The occurrence frequency of duration of humid heat events for light-rain and non-rainy cases over (60°S −60°N) in year 2001.

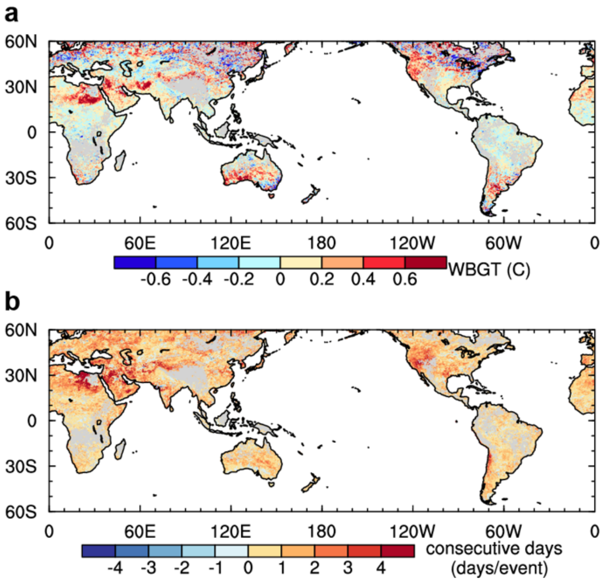

Supplementary Figure 9 | Intensity and duration of consecutive humid heat waves. Differences in the intensity (**a**) and duration (**b**) of consecutive events between light-rain and non-rainy cases.

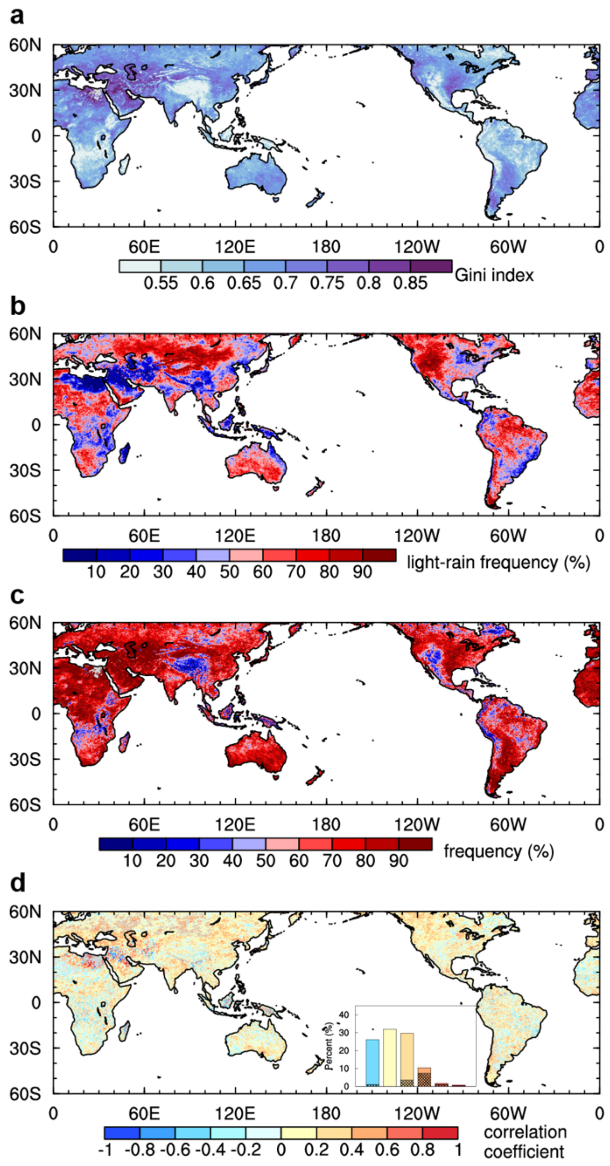

Supplementary Figure 10 | Gini index of precipitation, frequency of light rain and correlation between Gini index and wet bulb globe temperature (WBGT) intensity in consecutive humid heat waves. a, Gini index of precipitation during and the day before the consecutive events. **b,** Frequency of light rain during and the day before consecutive events. **c,** Among **b**, the relative frequency of one to three days with light rain. **d,** The correlation coefficient between the WBGT intensity of consecutive events and the corresponding Gini index, the bar chart shows the percentage of the coefficients in the intervals of ≤ 0, 0-0.2, 0.2-0.4, 0.4-0.6, 0.6-0.8 and 0.8-1.0, exceeding the 95% confidence level of the t-test are shaded.

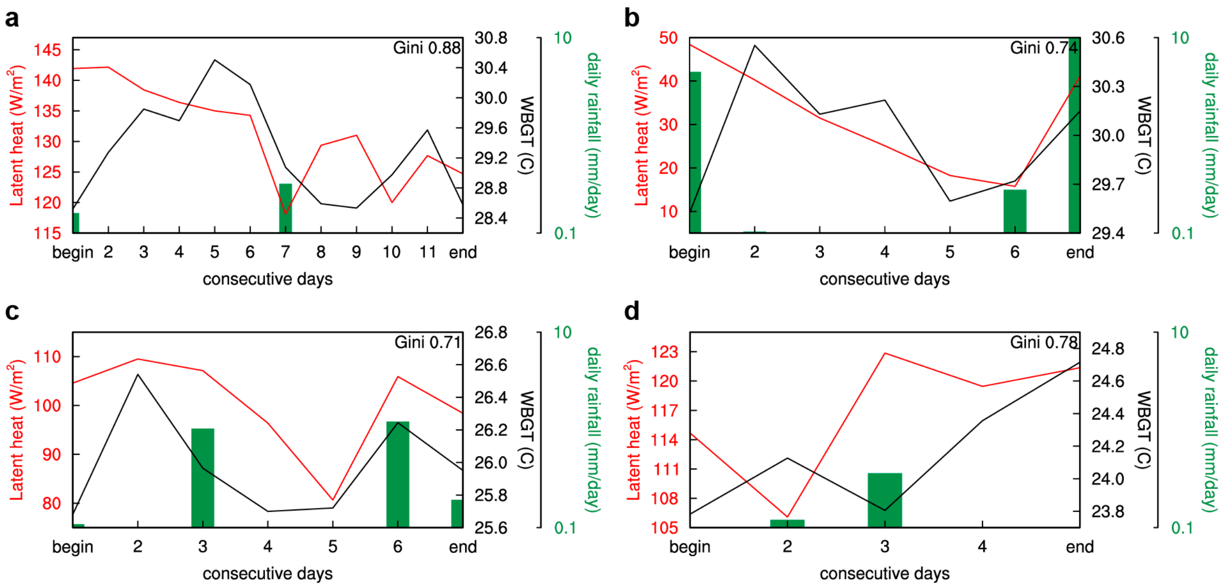

Supplementary Figure 11 | Latent heat flux and wet bulb globe temperature (WBGT) as functions of the day in consecutive events. The changes in latent heat flux and WBGT over time in a consecutive humid heat wave in the grid cells where Shanghai (**a**), New Delhi (**b**), Kinshasa (**c**), and Sao Paulo (**d**) are located, respectively. The bar denotes the daily amount of rainfall.

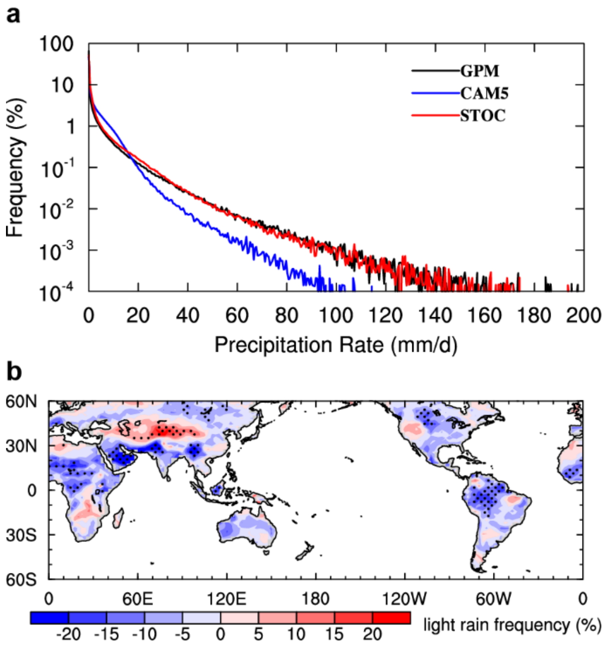

Supplementary Figure 12 | Frequency of rainfall in the hottest four months. a, The PDFs of rainfall intensity over land over (60°S–60°N). **b,** Differences between STOC and CAM5 (STOC minus CAM5) in simulating the light rain frequency.

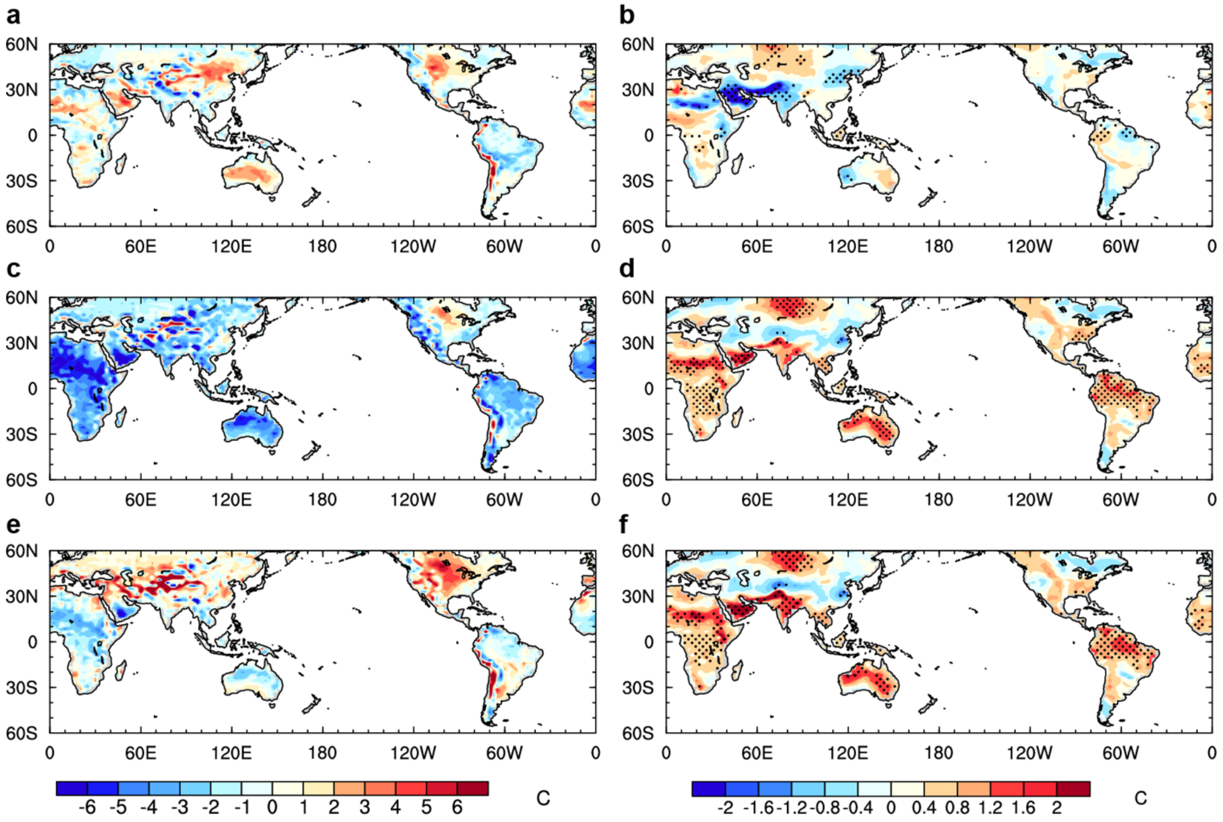

Supplementary Figure 13 | Model simulated wet bulb, black globe, and dry bulb temperatures. a, b, Difference in natural wet bulb temperature (T_w) between CAM5 simulation and ERA5 (**a**) and difference between STOC and CAM5 (**b**). **c, d,** Difference in black globe temperature (T_g) between CAM5 simulation and ERA5 (**c**) and the difference between STOC and CAM5 (**d**). **e, f,** Difference in dry bulb temperature (T_a) between CAM5 simulation and ERA5 (**e**) and the difference between STOC and CAM5 (**f**).

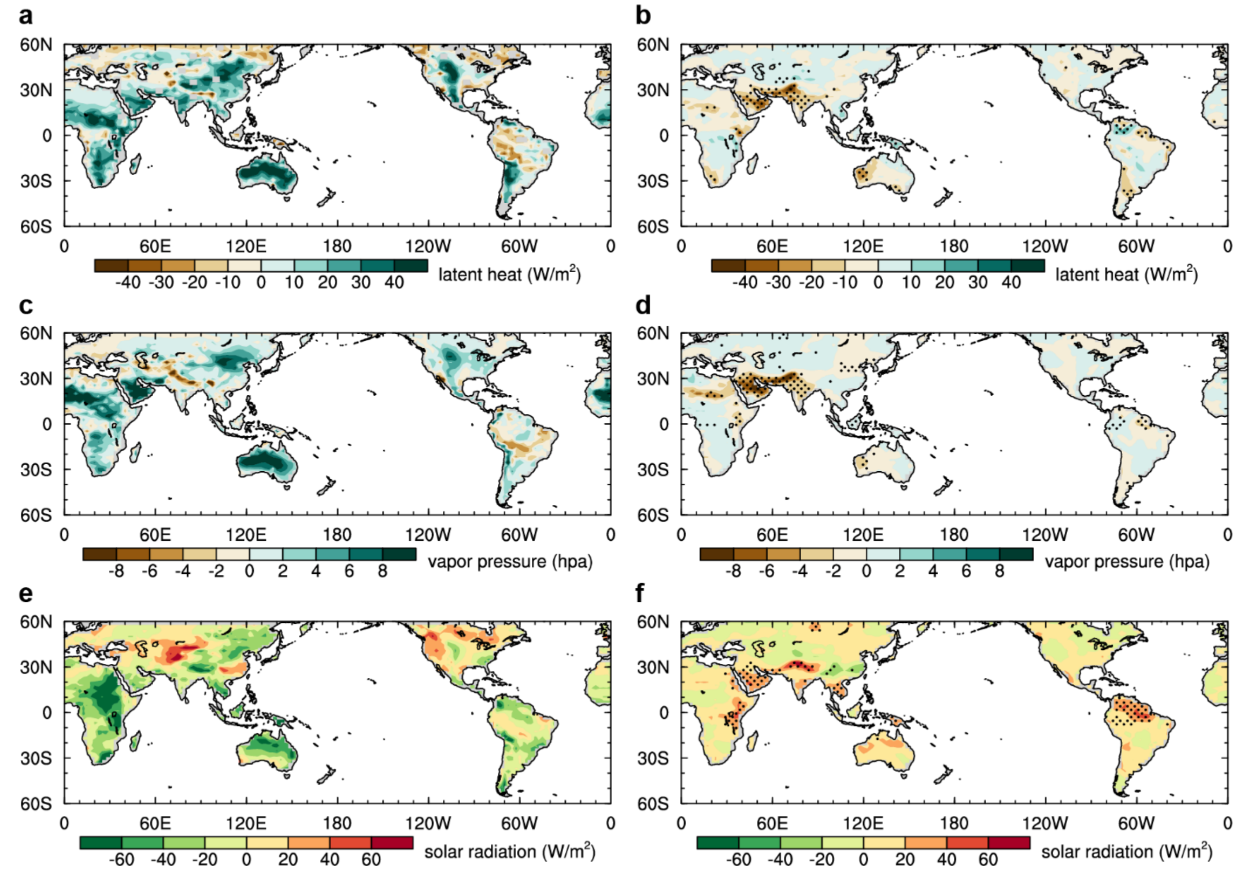

Supplementary Figure 14 | Model simulated surface latent heat flux, water vapor pressure, and downwelling shortwave radiation. a, b, Difference in surface latent heat flux between CAM5 simulation and FLUXCOM (**a**) and difference between STOC and CAM5 (**b**). **c, d,** Difference in surface vapor pressure between CAM5 simulation and ERA5 (**c**) and the difference between STOC and CAM5 (**d**). **e, f,** Difference in downwelling solar radiation between CAM5 simulation and CERES (**e**) and the difference between STOC and CAM5 (**f**).

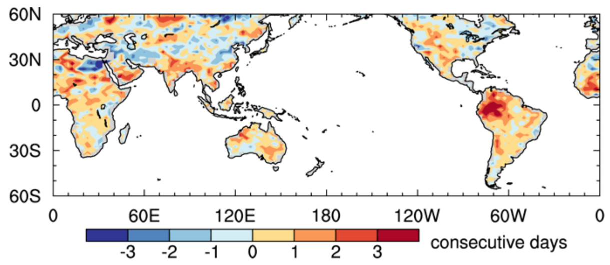

Supplementary Figure 15 | CAM5 and STOC simulation results. Differences between STOC and CAM5 (STOC minus CAM5) average duration of consecutive humid heat in the hottest four months.

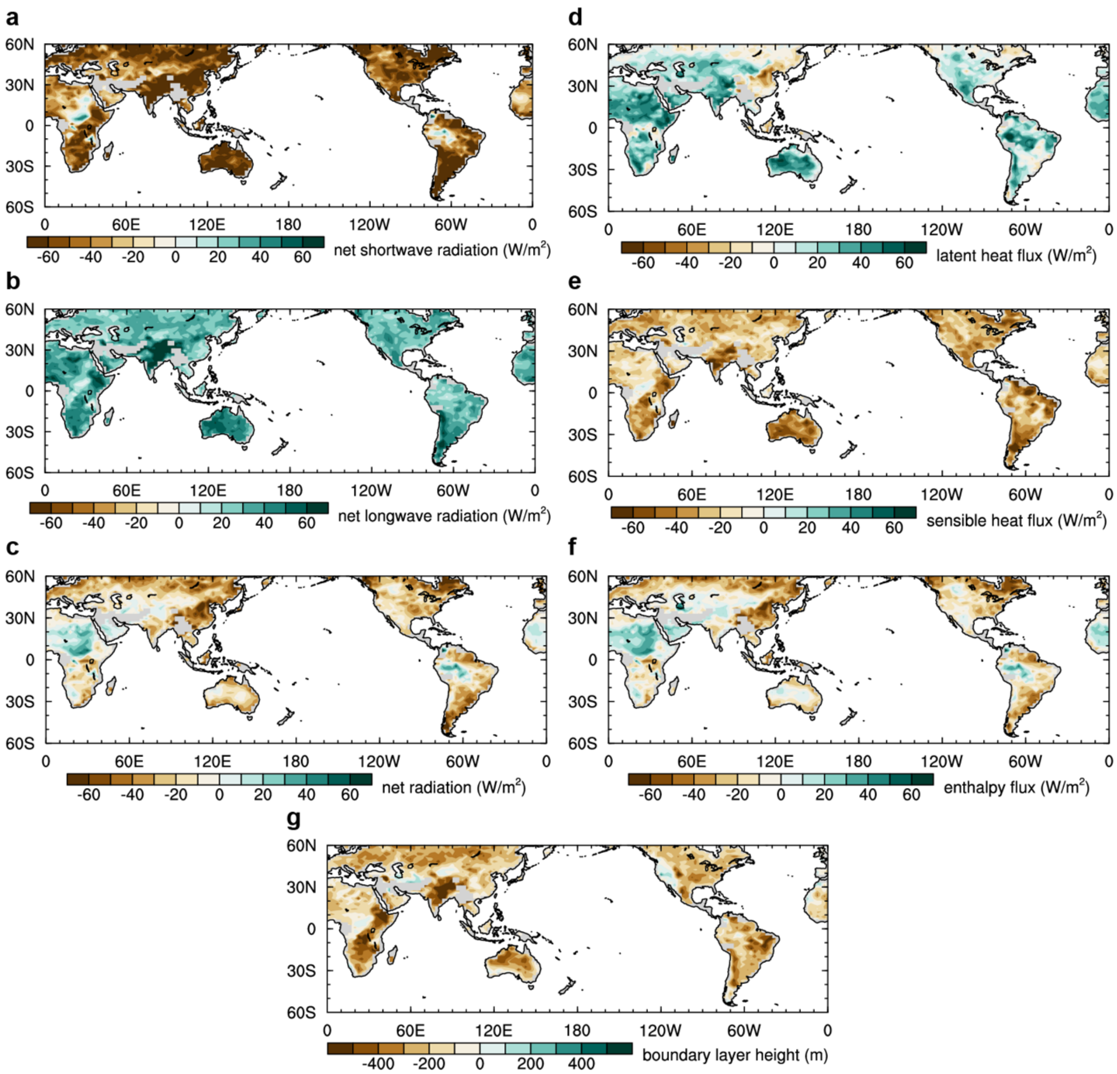

Supplementary Figure 16 | Differences in surface radiation, enthalpy flux and planetary boundary layer height between light rain and non-rainy days. Differences in surface net shortwave (**a**), net longwave (**b**), net total radiation (**c**), latent heat flux (**d**), sensible heat flux (**e**), total enthalpy flux (**f**), and planetary boundary layer height (**g**) between light rain days and non-rainy days (light rain days minus non-rainy days) in humid heat waves in the STOC simulation.

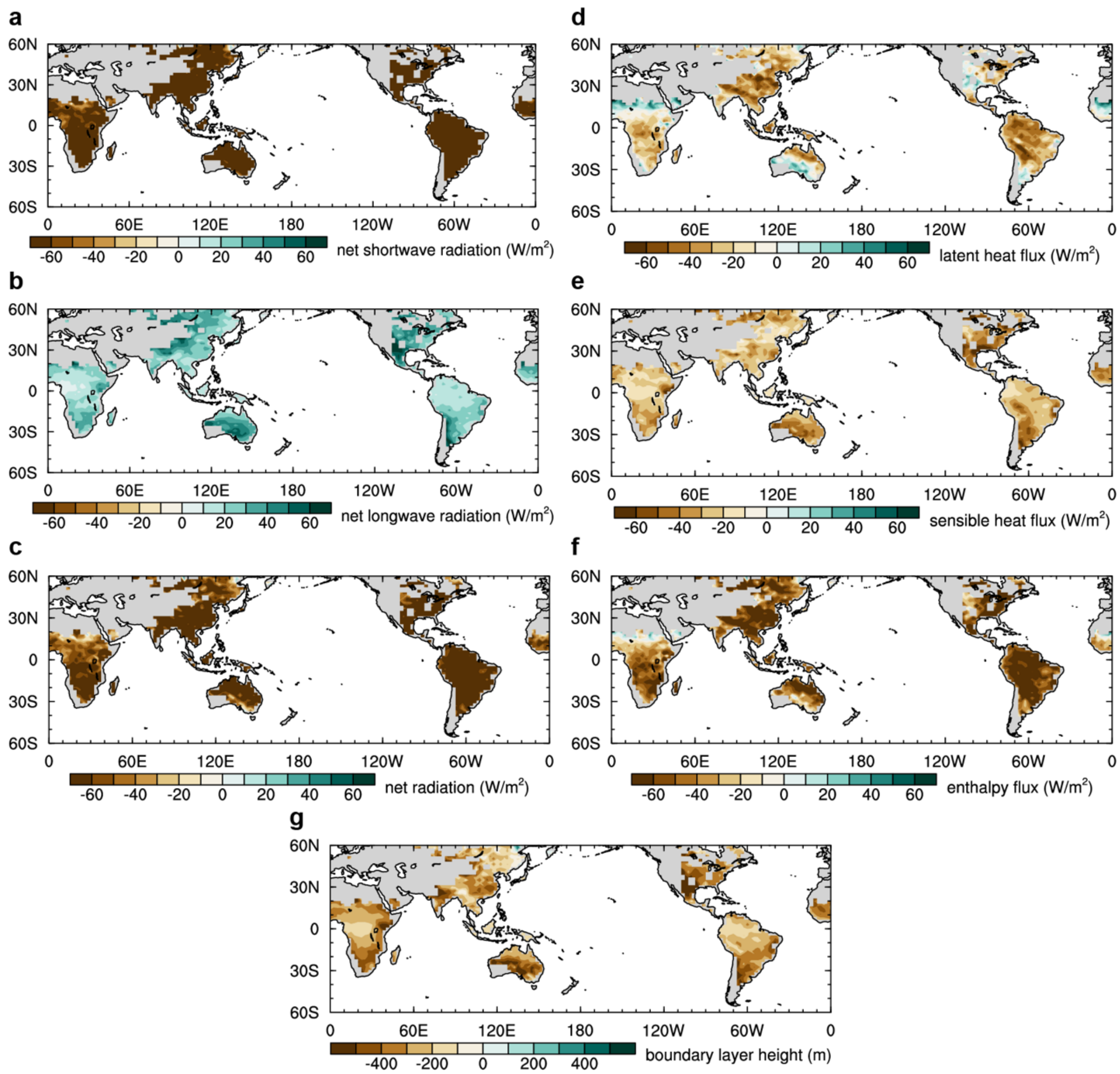

Supplementary Figure 17 | Differences in surface radiation, enthalpy flux and planetary boundary layer height between moderate-to-heavy rain days and light rain days. Differences in surface net shortwave (**a**), net longwave (**b**), net total radiation (**c**), latent heat flux (**d**), sensible heat flux (**e**), enthalpy flux (**f**), and planetary boundary layer height (**g**) between moderate-to-heavy rain days and light rain days (moderate-to-heavy rain days minus light rain days) in humid heat waves in the STOC simulation.

Supplementary Table 1 | Model Evaluation. Root mean squared error (RMSE) and correlation coefficient between CAM5/STOC simulated and observed light rain frequency, wet bulb globe temperature (WBGT), 0.7 times natural wet bulb temperature (T_w), 0.2 times black globe temperature (T_g), 0.1 times dry bulb temperature (T_a), surface latent heat flux (LH), near-surface vapor pressure (VP), downwelling solar radiation (DSW), Gini index and duration of consecutive events (WBGT95 days per event).

		Light rain frequency	WBGT	0.7Tw	0.2Tg	0.1Ta	LH	VP	DSW	Gini index	WBGT95 days per event
RMSE	CAM5	25.47	2.05	1.49	0.71	0.28	23.61	3.88	30.72	0.27	1.16
	STOC	21.40	2.03	1.49	0.64	0.28	21.80	3.67	26.41	0.19	1.30
COR	CAM5	0.73	0.94	0.92	0.93	0.92	0.76	0.86	0.80	0.31	0.27
	STOC	0.77	0.94	0.92	0.94	0.93	0.80	0.87	0.83	0.34	0.14
